# Latent Impulsivity Subtypes in Substance Use Disorders and Interactions with Internalizing and Externalizing Co-Occurring Disorders

**DOI:** 10.3389/fpsyt.2018.00027

**Published:** 2018-02-09

**Authors:** Rodrigo Marín-Navarrete, Aldebarán Toledo-Fernández, Luis Villalobos-Gallegos, Carlos Roncero, Nestor Szerman, María Elena Medina-Mora

**Affiliations:** ^1^National Institute of Psychiatry Ramón de la Fuente Muñiz, Mexico City, Mexico; ^2^Clinical Trials Unit on Addiction and Mental Health, National Institute of Psychiatry Ramón de la Fuente Muñiz, Mexico City, Mexico; ^3^Psychiatric Service, University of Salamanca Health Care Complex, Institute of Biomedicine of Salamanca (IBSAL), University of Salamanca, Salamanca, Spain; ^4^Spanish Society on Dual Disorders (SEPD), Madrid, Spain; ^5^Gregorio Marañón University Hospital, Madrid, Spain

**Keywords:** impulsivity, substance use disorders, co-occurring disorders, internalizing disorders, externalizing disorders, latent class analysis

## Abstract

This study explored the clinical importance of latent impulsivity subtypes within a sample of individuals with substance use disorders (SUDs) and high rates of co-occurring disorders (CODs) receiving residential treatment, aiming to assess the heterogeneity of the associations between SUDs and CODs across such impulsivity subtypes. The abbreviated Barratt impulsiveness scale was used to assess motor and cognitive (attentional and nonplanning) impulsivity, a structured interview for diagnosis of SUD and CODs, and other clinimetric measures for severity of substance use. Latent class analysis was conducted to extract subgroups of impulsivity subtypes and Poisson regression to analyze effects of interactions of classes by CODs on severity of substance use. 568 participants were evaluated. Results featured a four-class model as the best-fitted solution: overall high impulsivity (OHI); overall low impulsivity; high cognitive-low motor impulsivity; and moderate cognitive-low motor impulsivity (MC-LMI). OHI and MC-LMI concentrated on most of the individuals with CODs, and individuals within OHI and MC-LMI showed more severity of substance use. The expression of this severity relative to the impulsivity subtypes was modified by their interaction with internalizing and externalizing CODs in very heterogeneous ways. Our findings suggest that knowing either the presence of trait-based subtypes or CODs in individuals with SUDs is not enough to characterize clinical outcomes, and that the analysis of interactions between psychiatric categories and behavioral traits is necessary to better understand the expressions of psychiatric disorders.

## Introduction

Impulsivity is not a unitary construct; a well-founded body of evidence shows that it comprises several heterogeneous traits (1–3), which are helpful for describing and understanding different behavioral phenomena along the continuum from normality to pathology (4). A recurrent collection of impulsivity traits is thought to include lack of focus on ongoing task (attentional impulsivity), deficits of forethought (nonplanning impulsivity), and tendency to act without thinking (motor impulsivity) (4, 5).

Because these traits have been consistently associated with etiology and clinical outcomes of several psychiatric disorders, including substance use disorders (SUDs) and externalizing and internalizing co-occurring disorders (CODs) (4, 6, 7) [CODs: are defined as the co-occurrence or concomitance of SUDs with other psychiatric disorders (OPDs)], clinical conditions that may develop sequentially or in parallel across the lifespan (8), they are considered to be transdiagnostic constructs, plausible dimensions within the matrix of the Research Domain Criteria (9), as well as candidates for psychiatric endophenotypes (10–12). For example: in relation to bipolar disorder, variations in levels of impulsivity traits may characterize the different affective states of the disorder, with motor impulsivity levels higher at manic episodes, and attentional impulsivity higher both at manic and depressive episode; in turn, these variations of impulsivity may also be related to clinical outcomes such as suicide attempts (4).

However, many of the findings concerning the associations between impulsivity traits and CODs do not help to clarify their transdiagnostic nature. Published research tend to address the issue of impulsivity profiling by formation of groups based on levels of impulsivity derived from sum scores, or by linear associations between these sum scores of impulsivity and specific psychiatric variables, within usually small and single-diagnosed samples (4, 13, 14). As useful as these findings are, they assume that the associations between impulsivity traits and CODs remain homogeneous across individuals and disorders, with little concern about the probable combinations of impulsivity traits and their mediated expression relative to specific disorders; for instance, individuals with low motor and high attentional impulsivity could show distinct outcomes when compared to individuals with overall high impulsivity (OHI); furthermore; an individual with the former impulsivity profile also diagnosed with major depression might show different clinical outcomes relative to an individual with the same impulsivity profile diagnosed with an anxiety disorder. Thus, the common impulsivity findings do not account for the empirical recognition of the multiple interrelations of impulsivity traits across individuals, for the heterogeneous interactions between these patterns and psychiatric disorders, or for the effects of these interactions on the expression of multiple clinical outcomes.

A better approach to this problem is through the identification of impulsivity subtypes based on individuals’ combinatorial patterns of impulsivity traits. For this purpose, latent class analysis (LCA) is a suitable analytic approach. The LCA empirically unveils sets of underlying subgroups with similar patterns of response in relation to a given observable categorical variable. These latent subgroups further allow for a pragmatic characterization of respective clinical profiles, while controlling for methodological issues, such as high type I error rate, or low statistical power (15).

Few studies on impulsivity have been carried out using this analytical strategy and, to our knowledge, only one (16) in a sample of individuals with addictive behaviors and CODs. This study measured trait and cognitive (performance test) impulsivity in cocaine users and pathological gamblers, and identified two classes of impulsivity subgroups: one class characterized by greater trait impulsivity and poorer cognitive impulsivity, and another characterized by lower trait impulsivity and better cognitive impulsivity. Both classes showed different personality profiles and clinical outcomes. The study then compared between class differences in personality profiles (e.g., narcissistic, avoidant, schizoid) and clinical outcomes (e.g., craving, depression, somatic symptoms). However, the possible moderating effect of these subtypes in association between personality profiles and clinical outcomes was not analyzed, leaving us wondering about the possibility of the heterogeneity of the association between such variables (e.g., an impulsivity subtype characterized by greater trait impulsivity and poorer cognitive impulsivity may indeed express different levels of association between depression, craving, or somatic symptoms and narcissistic, schizoid, or avoidant traits when compared to a subtype of lower trait impulsivity and better cognitive impulsivity). Besides, the small sample size (*n* = 96) used in this study may have limited the information that LCA can deliver, since smaller samples tend to allow for extraction of fewer number of classes (17), to underrepresent classes with lower prevalence (18), and to compromise statistical power.

To address these issues, we evaluated individuals with polysubstance use in community-based residential care facilities, characterized by high prevalence of CODs (19, 20) with the ultimate goal of evaluating the heterogeneity of the associations between internalizing and externalizing CODs on severity of substance use across impulsivity subtypes. To accomplish this, we also carried out the following preliminary steps: (1) to determine the existence of latent impulsivity subtypes of individuals diagnosed with SUD, using LCA; (2) to describe clinical profiles for the different latent classes in two categories: presence of internalizing and externalizing CODs, and severity of substance use (e.g., age of onset, related problems).

## Materials and Methods

### Participants

The study comprised patients from 30 residential facilities for substance use treatment in five Mexican states (State of Mexico, Puebla, Queretaro, Hidalgo, and Mexico City). Inclusion criteria were: being between 18 and 60 years of age, being literate, having no less than 1 week since intake, and having conceded informed consent. These criteria were considered to control for bias due to cognitive deficits related to age, education, and acute substance intoxication, and to procure capacity for consent. Patients that endorsed a current psychotic, manic or hypomanic episode, or cognitive impairment were excluded, since previous evidence points out that these conditions compromise their ability to complete participation or to provide accurate and meaningful information as the result of cognitive bias (e.g., memory recall bias, rapid and overconfident judgments, and cognitive inflexibility) (21–24).

### Measures

#### Classification Measure

##### Abbreviated Barratt Impulsiveness Scale (ABIS)

It is a brief form of the Barratt Impulsiveness Scale-11^th^ version (BIS-11) obtained through confirmatory factor analysis with two sequential independent samples. The ABIS includes the following items from the BIS-11, grouped according to the traits they measure: attentional impulsivity: 5, 8, 9, 12, 20; motor impulsivity: 2, 14, 17, 19; nonplanning impulsivity: 1, 7, 13, and 30. These items ask the participant to score different impulsivity indicators on a four-point anchored scale (“Rarely/Never” = 1, “Occasionally” = 2, “Often” = 3, “Almost Always/Always” = 4). Values are reversed for items 1, 7, 8, 9, 12, 13, 20, and 30. The ABIS has comparative advantage over other proposed abbreviations of the BIS-11, because it retains the three-factor measurement model of impulsivity with acceptable goodness-of-fit (CFI = 0.97; RMSEA = 0.05[0.05, 0.06]) and similar reliability as the original scale (25). For this study, the English-version of the ABIS was adapted through a back-translation process.

#### Outcome Measures

##### CODs. Mini International Neuropsychiatric Interview-Fifth Version (MINI-5)

It is a structured interview used for rapid and accurate diagnosis of psychiatric disorders according to DSM-IV criteria (26). A Spanish-adapted version was used. Diagnostic results (presence or absence of disorder) from the interview modules for the following psychiatric conditions were considered: SUD (alcohol or drug dependence or abuse), depressive disorder (DD) (major DD or dysthymia), general anxiety disorder (GAD), post-traumatic stress disorder (PTSD), antisocial personality disorder (ASPD), attention deficit/hyperactivity disorder (ADHD), social phobia, and eating disorder (ED) (anorexia nervosa and bulimia nervosa).

##### Severity of Substance Use: Time of Substance Use

It included the following variables: (1) onset of substance use (*“How old were you when you start using* [alcohol, marihuana, inhalants, cocaine or other substances]?”); (2) longest time period of abstinence (*“Since you started to use* [substance], *what was your longest period of abstinence [in months]?”*); and (3) days substance use of 30 days before treatment admission: (*“How many of the 30 days before starting treatment did you use* [substance]?”). The later was used as a measure of severity of substance use within the main analysis. Items 1 and 3 were extracted from a Spanish version of the addiction severity index (27); item 2 was designed *ex professo* for this study.

##### Severity of Substance Use: Short Inventory of Problems-Revised (SIP-R)

It is a 15-item self-report measure to assess adverse consequences of alcohol and drug use in five domains: interpersonal, intrapersonal, physical, impulse control, and social. The SIP-R instructs participants to point out how often each of the listed consequences has occurred during the past 3 months (“Never” = 0, “Once or few times” = 1, “Two times a week” = 2, “Daily or almost daily” = 3). A five-factor model with a version of the SIP-R (28) showed acceptable goodness-of-fit (χ*^2^* = 708.03; df = 85; CFI = 94; RMSEA = 0.09). For this study, we used a Spanish-language version adapted in Hispanic Americans (29). This variable was also used as a measure of severity of substance use in the main analysis.

### Procedures

All individuals were recruited for participation at each facility after a group session with all resident patients to inform about the objectives and procedures of the study. Interested participants went through an individual informed consent process with an interviewer who provided more detailed information on study assessments, risks, benefits, and participant rights. Eligible participants completed the evaluation in one or two sessions. Exclusion criteria were checked using the Mini-Mental State Examination (30) for cognitive impairment, and the MINI-5 for current mania/hypomania or current psychotic disorder. In a second phase, to every eligible participant a battery that included several clinimetric measures, including the ABIS and the SIP-R, was administered. All assessment procedures were conducted by trained interviewers overseen by a field supervisor. All interviewers and supervisors were staff members from the local institutes and councils against addiction, had experience in addiction treatment, and completed at least a bachelor’s degree level of education in health sciences. All research team members went through a training process on study assessments and procedures delivered by two clinical experts (a psychiatrist and a psychologist). Training consisted of a 2-day centralized workshop and 6 post-training webinar sessions, while certification was achieved through a role-playing exercise.

### Ethical Considerations

All protocol procedures, informed consent forms, assessment forms, and patient recruitment materials were approved by the Research Ethics Committee of the National Institute of Psychiatry Ramón de la Fuente Muñiz, and adhered to the Declaration of Helsinki. For all participants who endorsed criteria for any psychotic, manic or hypomanic episode, or suicide behavior, the research team informed the site director and local health authorities in charge to ensure patients received specialized treatment.

All residential treatment facilities participating in the study were already certified by the ministries of health of each participating state prior to their involvement in the study. These facilities do not count with ethics committees; their directors were informed about all the procedures of the study as an ethical requisite demanded by the Research on Ethics Committee of the National Institute of Psychiatry Ramón de la Fuente Muñiz.

### Statistical Analysis

Since the first step to attain the objective of this study was to determine the best LCA model, we conducted the following procedure: ABIS items were included as ordered categorical indicators. Two- to six-class models were evaluated. Bayesian information criteria (BIC), sample adjusted-BIC (aBIC), class entropy, and bootstrapped likelihood ratio test (BLRT) were estimated for every class. To determine which model obtained the best comparative fit, we used the recommendations by Nylund et al. (18). The best fitting model had to obtain the lowest BIC, aBIC, and a significant BLRT (*p* < 0.05). To prevent local solutions every model was estimated with 1,500 random starts and 10 optimizations. We operationalized a global solution as the replication of the best log-likelihood in 10 from 10 optimizations. This analysis was performed using the statistical software Mplus 8 (31).

As the second step of the analysis, participants’ characteristics were described in relation to the total sample and to the extracted classes, and they comprised: gender, age, onset of substance use, longest period (in months) of abstinence, days of substance use during 30 days prior to admission in residential treatment, and the presence of the following psychiatric conditions: SUD (including: alcohol or drug dependence or abuse), any COD (including: DD, GAD, PTSD, ASPD, ADHD, social phobia, and EDs), internalizing CODs (including: DD, GAD, and PTSD), and externalizing CODs (including: ASPD and ADHD). Count and numeric variables are presented as mean (SD) and categorical variables as frequencies (percentages). Univariate comparisons of count and numeric variables between classes were carried out using one-way ANOVA and for categorical variables chi-square test. Two-sided *p* < 0.05 was considered significant. IBM SPSS version 23 was utilized for this analysis. Finally, to assess the heterogeneity in the associations between internalizing and externalizing CODs and severity of substance use, we extracted the most probable class, and then analyzed the moderation effect. For this purpose, independent models were tested using Poisson regression. Outcome variables for each model were substance use-related problems (SIP-R’s domains and total score), and days of substance use during 30 days prior to residential treatment. Predictor variables for both models were: most probable class membership, internalizing and externalizing CODs. Second-order interaction terms, class × externalizing CODs and class × internalizing CODs, were included in both models. This analytical approach is an analogous procedure applied to cross-sectional studies derived from the test of heterogeneity of effects in longitudinal research (32).

**Figure 1:**
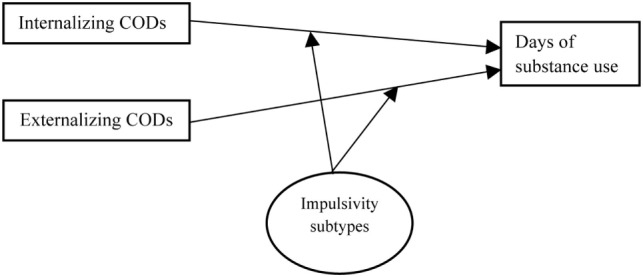
Graphical representation of the analytical model. In the first step, the categorical latent variable (Impulsivity subtypes) was estimated using LPA. In the second step, interactive terms (Internalizing disorders × impulsivity subtype and externalizing disorders × impulsivity subtype) were included and tested using Poisson regression, to determine the differential effect across impulsivity subtypes.

Age and gender were controlled as confounders. Significant *p*-value was set at <0.05. These analyses were performed with R version 3.4.2, using the glm() function (Figure 1).

## Results

### Descriptive Analysis for the Total Sample

A total of 657 participants were recruited; from these, seven failed to complete initial assessment, 49 did not endorse criteria for any SUD, and 33 were excluded due to cognitive impairment, current psychosis, or current mania or hypomania, leaving a total of 568 participants whom completed the evaluation with the ABIS. Most of the sample was male (88%) with a mean age of 30.37 (SD = 10.9; range = 18–60), and reported lifetime use of: alcohol (96.3%), cocaine (74.11%), marijuana (78.69%), and inhalants (47.88%) (Table 2). 92.3% of participants reported use of any substance in the past month prior to their admission to treatment. Use of methamphetamines, heroine, methadone, hallucinogens, and sedatives, was reported by about 2–5% of the sample, and, therefore, these substances were not considered in the main analysis.

### LCA and Descriptive Analysis by Classes

Table 1 displays fit measures for two- to six-class models. The four-class model obtained lower AIC, BIC, and aBIC values compared to models with lesser number of classes, and replicated 10 times of 10 optimizations, in contrast with five- and six-class models which, despite obtaining higher fit values, did not reach full replications, and thus such solutions were considered local.

**Table 1:** Fit indices for the different classes.

Model	AIC	BIC	aBIC	Entropy	BLRT^a^
Class 1	19353.303	19522.645	19398.838	n/a	
Class 2	18451.849	18794.876	18544.088	0.806	0.000
Class 3	17920.268	18436.981	18059.210	0.841	0.000
**Class 4**	**17647.276**	**18337.674**	**17832.922**	**0.865**	**0.000**
Class 5^b^	17467.198	18331.280	17699.547	0.870	0.000
Class 6^b^	17342.624	18380.391	17621.676	0.877	0.000

*^a^p value*.

*^b^Failed to attain the global solution criteria*.

*Best solution is displayed in bold letters. AIC, Akaike Information Criterion; BIC, Bayesian Information Criterion; aBIC, Sample-Size Adjusted BIC; BLRT, Bootstrap Likelihood Ratio Test*.

**Table 2:** Characteristics of participants for each of the classes.

	Total (*n* = 568) *n* (%) or$\bar{x}$(SD)	OHI (*n* = 156) *n* (%) or$\bar{x}$(SD)	OLI (*n* = 105) *n* (%) or$\bar{x}$(SD)	HC-LMI (*n* = 67) *n* (%) or$\bar{x}$(SD)	MC-LMI (*n* = 240) *n* (%) or$\bar{x}$(SD)	Statistical differences between classes
Sex						
Male	500 (88.02)	128 (82.05)	96 (91.42)	61 (91.04)	215 (89.58)	χ^2^ = 7,569(3), *p* = 056
Female	68 (11.97)	28 (17.94)	9 (8.57)	6 (8.95)	25 (10.41)	
Age	30.37 (10.90)	25.42 (8.04)	33.07 (11.57)	29.97 (11.44)	32.53 (11.03)	*F*(3,564) = 17.41, *p* < 001
Alcohol^a^	547 (96.30)	152 (97.43)	98 (93.33)	67 (100)	230 (95.83)	
Onset	17.46 (6.21)	15.93 (3.80)	18.70 (6.54)	15.94 (4.88)	18.38 (7.33)	*F*(3,543) = 7.66, *p* < 001
Abstinence^b^	21.96 (43.70)	14.36 (25.36)	29.17 (53.59)	20.82 (34.20)	24.23 (50.10)	*F*(3,543) = 2.66, *p* = 047
Use past 30 days^c^	9.75 (10.41)	9.22 (9.89)	8.20 (10.15)	12.07 (11.31)	10.08 (10.50)	*F*(3,543) = 2.05, *p* = 106
Cocaine^a^	421 (74.11)	126 (80.76)	76 (72.38)	46 (68.85)	173 (72.08)	
Onset	19.72 (5.70)	18.56 (3.36)	22.37 (7.66)	17.65 (5.17)	19.95 (5.77)	*F*(3,417) = 9.90, *p* < 001
Abstinence^b^	39.66 (59.38)	21.66 (42.09)	51.43 (69.50)	34.33 (50.77)	49.02 (64.40)	*F*(3,417) = 6.67, *p* < 001
Use past 30 days^c^	7.39 (12.80)	8.06 (11.15)	7.05 (19.08)	6.83 (10.56)	7.20 (11.02)	*F*(3,417) = 17, *p* = 915
Marijuana^a^	447 (78.69)	139 (89.10)	72 (68.57)	54 (80.59)	182 (75.83)	
Onset	16.87 (4.92)	16.96 (5.09)	17.83 (6.66)	16.19 (4.10)	16.64 (4.11)	*F*(3,443) = 1.42, *p* = 236
Abstinence^b^	48.48 (83.39)	28.93 (61.03)^d^	49.42 (73.70)	68.89 (107.38)	56.87 (91.02)	*F*(3,443) = 4.31, *p* = 005
Use past 30 days^c^	13.48 (38.80)	14.02 (13.94)	19.0 (91.73)	11.96 (14.14)	11.32 (13.59)	*F*(3,443) = 70, *p* = 548
Inhalants^a^	272 (47.88)	89 (57.05)	45 (42.85)	29 (43.28)	109 (45.41)	
Onset	17.36 (5.75)	17.38 (5.99)	17.60 (5.52)	16.07 (3.73)	17.60 (6.11)	*F*(3,268) = 0.57, *p* = 635
Abstinence^b^	57.44 (85.56)	42.21 (77.76)	59.09 (97.09)	53.72 (72.47)	70.17 (88.77)	*F*(3,268) = 1.78, *p* = 151
Use past 30 days^c^	12.51 (87.55)	7.27 (10.45)	8.71 (12.31)	9.31 (11.86)	19.20 (137.73)	*F*(3,268) = 35, *p* = 784
Substance use-related problems						
Total	29.25 (13.51)	32.81 (12.95)	27.55 (13.10)	27.75 (13.65)	28.09 (13.67)	*F*(3,564) = 5.14, *p* = 002
Physical	5.09 (2.71)	5.42 (2.66)	5.01 (2.67)	4.85 (2.66)	4.98 (2.78)	*F*(3,564) = 1.07, *p* = 357
Interpersonal	5.98 (2.87)	6.51 (2.80)	5.73 (2.88)	5.79 (3.03)	5.80 (2.84)	*F*(3,564) = 2.47, *p* = 061
Intrapersonal	5.17 (2.59)	5.61 (2.51)	5.06 (2.56)	4.73 (2.56)	5.05 (2.63)	*F*(3,564) = 2.39, *p* = 068
Impulsivity	3.08 (1.95)	3.68 (1.91)	2.77 (1.90)	2.71 (1.95)	2.93 (1.92)	*F*(3,564) = 7.32, *p* = 000
Social	5.72 (2.71)	6.35 (2.63)	5.44 (2.64)	5.52 (2.62)	5.50 (2.77)	*F*(3,564) = 3.86, *p* = 009

*^a^Individuals who reported lifetime use*.

*^b^Longest period (in months) of abstinence in lifetime*.

*^c^30 days prior to admission in residential treatment*.

*^d^1 missing value*.

*C, class; HC-LMI, high cognitive-low motor impulsivity; MC-LMI, moderate cognitive-low motor impulsivity; OHI, overall high impulsivity; OLI, overall low impulsivity*.

**Figure 2:**
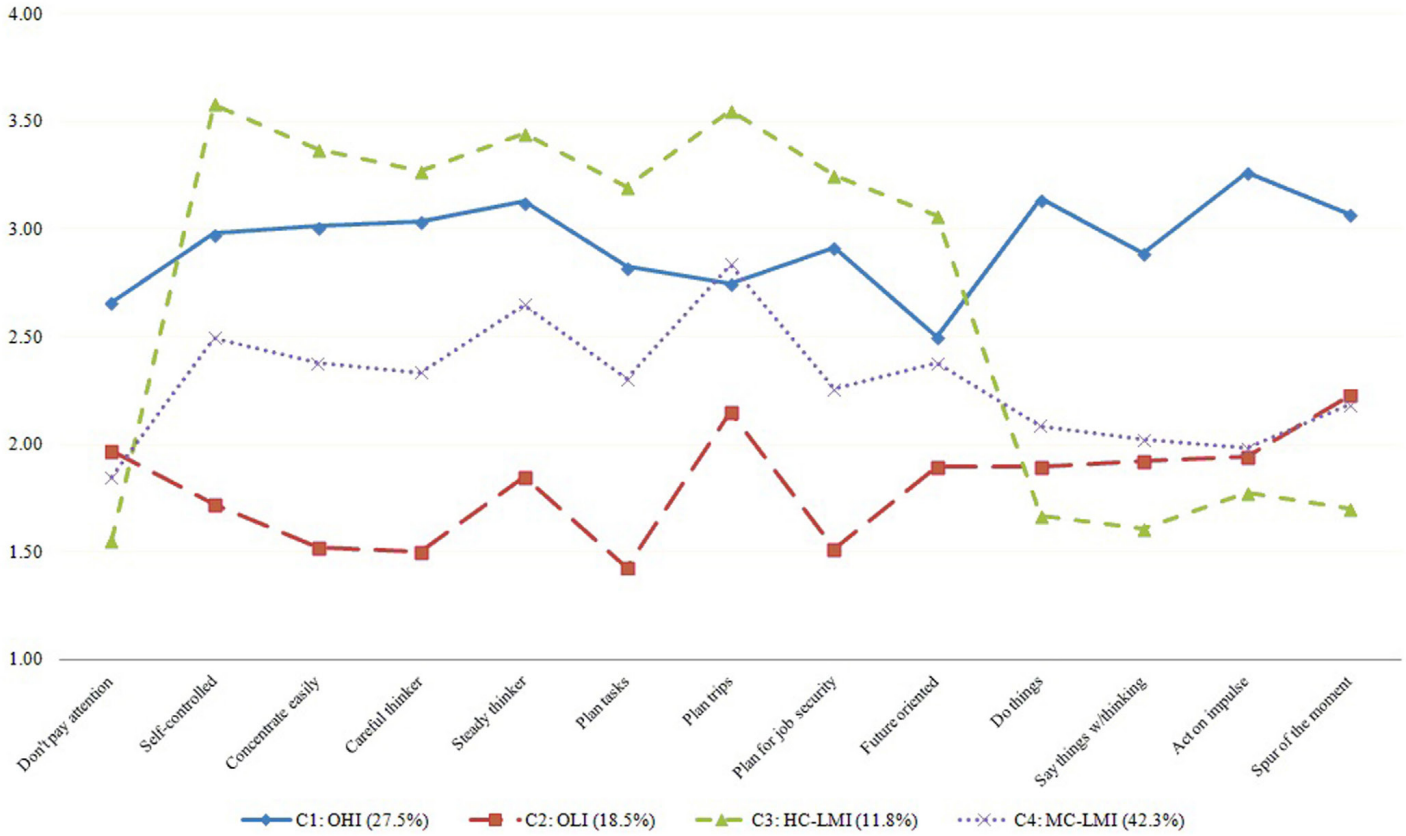
Latent classes of impulsivity in polysubstance users in residential treatment. Notes: *n* = 568. Values are presented after correction for inverted response options (e. g., “I concentrate easily”), so they indicate unidirectional level of impulsivity. C, class; OHI, overall high impulsivity; HC-LMI, high cognitive-low motor impulsivity; MC-LMI, moderate cognitive-low motor impulsivity; OLI, overall low impulsivity.

The four-class model was also retained in favor of theoretical parsimony, since clear recognizable patterns related to impulsivity traits could be identified (Figure 2). We labeled them as follows: based on combinations and levels of cognitive (attentional and nonplanning) and motor impulsivity: class 1: OHI; class 2: overall low impulsivity (OLI); class 3: high cognitive-low motor impulsivity (HC-LMI); and class 4: moderate cognitive-low motor impulsivity (MC-LMI).

The youngest participants were distributed within OHI and HC-LMI classes. Univariate differences between the four classes with regards to substance use were observed for onset of alcohol and cocaine use, and for longest period of abstinence of alcohol, cocaine, and marijuana, and SIP-R’s total score, impulsivity, and social domains (Table 1). Concerning the distribution of CODs, differences between the classes were noted for almost all psychiatric conditions, particularly: DD, PTSD, ASPD, ADHD, social phobia, and EDs, as well as for IDs and EDs, with higher prevalences within OHI and MC-LMI classes (Table 3).

**Table 3:** Distribution of CODs for each of the classes.

	OHI (*n* = 156) *n* (%)	OLI (*n* = 105) *n* (%)	HC-LMI (*n* = 67) *n* (%)	MC-LMI (*n* = 240) *n* (%)	Statistical differences
DD	74 (47.4)	22 (20.9)	28 (41.8)	65 (27.1)	χ^2^ = 27.60(3), *p* < 0.001
GAD	23 (14.7)	7 (66.7)	8 (11.9)	18 (7.5)	χ^2^ = 7.22(3), *p* = 0.065
PTSD	17 (10.9)	5 (47.6)	7 (10.4)	19 (79.2)	χ^2^ = 4.84(6), *p* = 0.564
ASPD	94 (60.2)	26 (24.8)	32 (47.9)	82 (34.2)	χ^2^ = 41.18(3), *p* < 0.001
ADHD	40 (25.7)	6 (57.1)	7 (10.4)	18 (7.5)	χ^2^ = 34.79(3), *p* < 0.001
Social phobia	21 (13.4)	7 (66.7)	8 (11.9)	14 (58.3)	χ^2^ = 8.27(3), *p* = 0.041
Eating disorder	13 (83.3)	0 (0)	7 (10.4)	5 (20.8)	χ^2^ = 19.452(3), *p* < 0.001
Internalizing CODs	93 (59.6)	29 (27.6)	39 (58.2)	79 (32.9)	χ^2^ = 44.054(3), *p* < 0.001
Externalizing CODs	101 (64.7)	27 (25.7)	33 (49.3)	85 (35.4)	χ^2^ = 49.484(3), *p* < 0.001
Any COD	125 (80.1)	43 (40.9)	49 (73.1)	122 (50.8)	χ^2^ = 55.25(3), *p* < 0.001

*C, class; CODs, co-occurring disorders; DD, depressive disorder; GAD, general anxiety disorder; HC-LMI, high cognitive-low motor impulsivity; MC-LMI, moderate cognitive-low motor impulsivity; OHI, overall high impulsivity; OLI, overall low impulsivity; PTSD, posttraumatic stress disorder; ED, eating disorder*.

### Regression Models

Using the OLI class as the category of reference and controlling for age and gender, significant main effects of class membership for substance use-related problems and substance use 30 days prior to treatment were observed. Specifically, scores of impulsivity and social domains of the SIP-R tended to increase relative to OHI class. Negative slopes were found for almost all of the SIP-R domains in association with HC-LMI class and for total score in association with MC-LMI class (Table 4). Days of alcohol use tended to increase in relation to all three classes, and negative slopes for days of cocaine, marijuana, and inhalant use were observed relative to HC-LMI class (Table 5).

**Table 4:** Class by CODs interactions with substance use-related problems.

	Total *B* (SE)	Intrapersonal *B* (SE)	Physical *B* (SE)	Interpersonal *B* (SE)	Impulsivity *B* (SE)	Social *B* (SE)
OHI	0.165 (0.034)*	0.131 (0.080)	0.029 (0.083)	0.124 (0.075)	0.328 (0.090)*	0.213 (0.062)*
HC-LMI	−0.229 (0.045)*	−0.258 (0.106)*	−0.253 (0.107)*	−0.187 (0.097)	−0.310 (0.128)*	−0.194 (0.083)*
MC-LMI	−0.026 (0.027)	−0.039 (0.064)	−0.038 (0.065)	−0.025 (0.060)	−0.028 (0.077)	−0.014 (0.051)
Internalizing CODs	0.176 (0.051)*	0.190 (0.120)	0.202 (0.119)	0.091 (0.116)	0.317 (0.130)*	0.138 (0.097)
Externalizing CODs	−0.051 (0.044)*	−0.129 (0.106)	−0.043 (0.103)	−0.088 (0.098)	0.138 (0.113)	−0.080 (0.082)
OHI × internalizing	−0.066 (0.059)	−0.083 (0.140)	−0.036 (0.139)	−0.064 (0.134)	−0.124 (0.149)	−0.047 (0.111)
HC-LMI × internalizing	−0.124 (0.059)*	0.203 (0.165)	0.141 (0.164)	0.184 (0.155)	0.271 (0.178)	0.281 (0.129)*
MC-LMI × internalizing	0.218 (0.069)*	−0.149 (0.141)	−0.101 (0.140)	−0.082 (0.135)	−0.201 (0.153)	−0.110 (0.113)
OHI × externalizing	0.091 (0.053)	0.119 (0.128)	0.159 (0.128)	0.115 (0.119)	−0.128 (0.136)	0.129 (0.099)
HC-LMI × externalizing	0.251 (0.064)*	0.236 (0.154)	0.286 (0.152)	0.267 (0.141)	0.239 (0.167)	0.237 (0.118)*
MC-LMI × externalizing	0.198 (0.050)*	0.219 (0.122)	0.152 (0.120)	0.169 (0.113)	0.175 (0.131)	0.248 (0.094)*

*All models are age- and gender-adjusted. Class 2 (overall low impulsivity) is the category of reference*.

*p < 0.05.

*C, class; CODs, co-occurring disorders; HC-LMI, high cognitive-low motor impulsivity; MC-LMI, moderate cognitive-low motor impulsivity; OHI, overall high impulsivity*.

**Table 5:** Class by CODs interactions with days of substance use past 30 days.

	Alcohol *B* (SE)	Cocaine *B* (SE)	Marijuana *B* (SE)	Inhalants *B* (SE)
OHI	0.361 (0.069)*	−0.067 (0.086)	−0.839 (0.052)*	0.050 (0.103)
HC-LMI	0.415 (0.075)*	−0.324 (0.115)*	−1.426 (0.090)*	−0.637 (0.162)*
MC-LMI	0.284 (0.054)*	−0.126 (0.069)	−1.175 (0.046)*	−0.151 (0.093)
Internalizing CODs	0.941 (0.079)*	0.687 (0.102)*	−0.319 (0.086)*	0.189 (0.126)
Externalizing CODs	0.129 (0.080)	0.069 (0.097)	−1.158 (0.076)*	0.783 (0.103)*
OHI × internalizing	−1.014 (0.097)*	−0.359 (0.120)*	0.315 (0.098)*	−0.047 (0.151)
HC-LMI × internalizing	−0.744 (0.107)*	−0.207 (0.152)	0.606 (0.117)*	0.334 (0.176)
MC-LMI × internalizing	−0.856 (0.095)*	−0.751 (0.124)*	0.567 (0.099)*	−1.452 (0.145)*
OHI × externalizing	0.179 (0.099)	0.329 (0.122)*	1.253 (0.091)*	−0.707 (0.134)*
HC-LMI × externalizing	0.362 (0.107)*	0.249 (0.150)	1.775 (0.119)*	0.240 (0.182)
MC-LMI × externalizing	0.322 (0.090)*	0.625 (0.112)*	1.618 (0.089)*	1.554 (0.121)*

*All models are age- and gender-adjusted. Class 2 (overall low impulsivity) is the category of reference*.

*p < 0.05.

*C, class; CODs, co-occurring disorders; HC-LMI, high cognitive-low motor impulsivity; MC-LMI, moderate cognitive-low motor impulsivity; OHI, overall high impulsivity*.

Relative to frequency of internalizing CODs, positive slopes were found for impulsivity and total scores of the SIP-R, and for days of alcohol and cocaine use; days of marijuana use showed negative association with this category. In relation to frequency of externalizing CODs, negative slopes were found for SIP-R total score and for days of marijuana use, days of inhalants use showed positive association with this category (Tables 4 and 5).

Concerning regression analyses of second-order interactions of classes by CODs (Tables 4 and 5), outcomes of severity of substance use were modified in several ways: (a) by establishing significant effects (e.g., MC-LMI by externalizing CODs revealed a positive slope for SIP-R social domain, and OHI by internalizing CODs revealed a negative slope for days of cocaine use); (b) by suppressing effects (e.g., interaction with externalizing CODs canceled statistical significance of OHI on days of alcohol use, and HC-LMI by internalizing CODs canceled significant effect on days of cocaine use); (c) by increasing effects (e.g., interaction with externalizing CODs increased the effect of HC-LMI on SIP-R total score); and (d) by changing the direction of the effects (e.g., interaction with internalizing CODs negatively changed the effect of all three classes on days of alcohol use).

## Discussion

This study had the main purpose to test the heterogeneity of the associations between internalizing and externalizing CODs across the subtypes of impulsivity. To reach this goal, we first had to determine the existence of latent impulsivity subtypes in the sample and to draw a comprehensive psychiatric profile relative to resulting impulsivity subtypes. Results featured a four-class model as the best-fitted solution. According to combinations and levels of cognitive (attentional and nonplanning) and motor traits, the impulsivity subtypes were labeled as OHI (individuals with SUD characterized by high deficits in attention and forethought, and high tendency to act without thinking), OLI (individuals with SUD characterized by low deficits in attention and forethought, and low tendency to act without thinking), HC-LMI (individuals with SUD characterized by slightly higher cognitive deficits than the OHI subgroup, and slightly lower motor impulsivity than the OLI subgroup), and MC-LMI (individuals with SUD characterized by deficits of cognitive impulsivity in-between OHI and OLI subgroups, and levels of motor impulsivity similar to the latter). It is to notice that, despite having a sufficiently clear pattern relative to the other classes, the HC-LMI class plotted a very low value for the item “I don’t pay attention,” regardless of its semantic similarity with the item “I concentrate easily,” which was found high within this class. Two explanations could be considered at the base of this tendency: (1) conservation of the capacity to initiate attentional behavior despite deficits to sustain it, or (2) a particular understanding of the item within this class rather than a differentiated behavioral trait.

In comparison to the OLI and MC-LMI subtypes, individuals conforming the OHI and HC-LMI subtype showed earlier onset of cocaine and alcohol. The OHI subtype proved more substance-use related problems, shorter periods of abstinence for cocaine and marijuana, and a greater proportion of internalizing and externalizing CODs followed by the MC-LMI subtype. Overall, deficits in attention and forethought appear to underline these clinical profiles, since HC-LMI and MC-LMI subtypes proved associations with severity of substance use and most of the studied psychiatric disorders (with the exception of anxiety disorders) independent of their individuals’ low endorsement of motor impulsivity traits. Rather, this motor component appears to boost these associations, as signaled by the OHI subtype. This observation is somewhat in line with findings linking cognitive impulsivity to cocaine and heroin use, with special involvement of motor impulsivity only for the case of the stimulant substance (33, 34). However, the observation is also at odds with other studies, reporting no significant association of motor impulsivity with alcohol (35) and cocaine use (36), though the discrepancy between methods restricts comparisons.

Furthermore, our results showed that the effects of impulsivity subtypes on severity of substance use are significantly modified by their interaction with internalizing or externalizing CODs in very complex ways. All classes proved to boost alcohol use and to diminish marijuana use; however, when interacting with internalizing CODs, reduced alcohol use, but increased marijuana use was observed, and so were increases in use of both substances when classes interacted with externalizing CODs. Interaction with internalizing CODs also worked as a reducer of cocaine use for the OHI and MC-LMI classes, and of inhalant use for the later, whereas interaction with externalizing CODs resulted in increased use of cocaine for the OHI and MC-LMI classes and of inhalants for the later. Substance use-related problems were also conditioned by these interactions, although to a lesser degree. OHI increased social and impulsivity problems, whereas HC-LMI proved to reduce all substance use-related problems, but these effects disappear for the former when interacting with internalizing or externalizing disorders, and changed direction of the effect for HC-LMI when interacting with externalizing disorders.

Though difficult as it is to parsimoniously characterize these associations, they have important implications. For instance, they challenge the general assumption of linearity between impulsivity and use of substances (4), rather suggesting that different trait-based, intra-homogeneous, and inter-heterogeneous impulsivity subtypes associate with distinctive patterns of use, in such a way that some subtypes could function as risk or protective factors for particular substances (e.g., HC-LMI for marijuana, cocaine, and inhalants, and all classes for alcohol, respectively). Also, being this association further modified by presence of CODs, it highlights the transdiagnostic nature of the impulsivity subtypes, suggesting that they may play several determinant roles in the differentiated expression of outcomes related to all prevalent CODs among populations of substance users, even with regards to disorders not essentially defined by impulsivity, such as internalizing disorders.

There are not many studies addressing the existence of impulsivity subtypes in population of substance users as to allow comparisons. The study by Albein-Urios et al. (16) reported a dichotomous solution describing a subgroup with lower cognitive- (measured with a behavioral test of inhibition) and higher trait impulsivity (measured with a self-report scale), and a subgroup with inverse characteristics, with the former showing greater levels of psychosocial dysfunction. This LCA solution, nonetheless, sheds more light over the dissociation between self-report measures of personality traits and behavioral procedures, which are frequently uncorrelated (4), than over subtypes of impulsivity traits. Considering the cognitive and motor components of impulsivity, the closest resemblance to our LCA findings appears to be the distinction between ADHD subtypes: inattentive, hyperactive-impulsive, and combined, which have been heterogeneously confirmed through LCA (37, 38). Studies associating these subtypes to OPDs have reported mixed findings: combined subtype is more strongly related with externalizing disorders; ADHD-inattentive symptoms predict onset, frequency, and quantity of use of several substances (39); ADHD-hyperactive symptoms predict internalizing problems, variety of used substances, and dependence symptoms (40). However, none of these studies utilized LCA to determine the ADHD subtypes.

There are limitations to be recognized in this study. First, while our sample was not small relative to other studies on impulsivity (4), the subdivision of classes could have limited the statistical power to analyze the interaction of subtypes with CODs and substance use-related problems. We tried to avoid further subdivision of the sample by grouping externalizing and internalizing CODs for the regression analyses, at the same time preventing overcomplexity of the results. This grouping signals a second limitation of the study, since both categories of CODs are not mutually exclusive within individuals, and in fact were commonly concurrent. Effects of multicollinearity should be taken into account in this regard when pondering the findings, since all participants were diagnosed with SUDs and frequently another externalizing disorder (both conditions defined by impulsivity) even within the group of participants with internalizing CODs. Third, our sample included only substance users in residential treatment, thus rendering uncertain whether the results may generalize to other populations of users. Last, when pondering our findings, it is important to recognize the inherent limitations of self-reports and interviews that rely on participants’ capacity for insight and for recalling of distal experiences (e.g., specific behaviors at elementary school), which, when compromised, could be an important source of bias, especially in individuals with SUDs and OPDs associated to cognitive dysfunction (23, 41). We tried to temperate these limitations by careful exclusion of individuals at risk of cognitive impairment, and by selecting measures with proven psychometric validity in these populations.

This study is an effort to identify latent impulsivity subtypes within population of substance users. Beyond this construct, our findings suggest that knowing either the presence of trait-based subtypes or CODs in individuals with SUDs is not enough to characterize clinical outcomes, and that the analysis of interactions between psychiatric categories and behavioral traits is necessary to better understand the expressions of psychiatric disorders.

## Ethics Statement

All protocol procedures, informed consent forms, assessment forms, and patient recruitment materials were approved by the Research on Ethics Committee of the National Institute of Psychiatry Ramón de la Fuente Muñiz, and adhered to the Declaration of Helsinki. For all participants who endorsed criteria for any psychotic, manic or hypomanic episode, or suicide behavior, the research team informed the site director and local health authorities in charge to ensure patients received specialized treatment.

## Author Contributions

RM-N conceptualized the study, established cooperation with the participating institutions, raised funding and, along with AT-F, conceptualized and wrote the article. LV-G conducted statistical analyses. CR, NS, and MM-M performed critical review of all the versions of the article. All authors have approved the current version of the article.

## Conflict of Interest Statement

The authors declare that the research was conducted in the absence of any commercial or financial relationships that could be constructed as a potential conflict of interest.
